# A Population-Based Study on Alcohol and High-Risk Sexual Behaviors in Botswana

**DOI:** 10.1371/journal.pmed.0030392

**Published:** 2006-10-10

**Authors:** Sheri D Weiser, Karen Leiter, Michele Heisler, Willi McFarland, Fiona Percy-de Korte, Sonya M DeMonner, Sheila Tlou, Nthabiseng Phaladze, Vincent Iacopino, David R Bangsberg

**Affiliations:** 1 Physicians for Human Rights, Cambridge, Massachusetts, United States of America; 2 Center for AIDS Prevention Studies, University of California San Francisco, San Francisco, California, United States of America; 3 Epidemiology and Prevention Interventions Center, Division of Infectious Diseases, San Francisco General Hospital, University of California San Francisco, San Francisco, California, United States of America; Veterans Affairs Ann Arbor Health System and Department of Internal Medicine, University of Michigan School of Medicine, Ann Arbor, Michigan, United States of America; 5 San Francisco Department of Public Health, San Francisco, California, United States of America; 6 Department of Nursing, University of Botswana, Gaborone, Botswana; 7 Department of Medicine, University of Minnesota, Minneapolis, Minnesota, United States of America; 8 Positive Health Program, San Francisco General Hospital, University of California San Francisco, San Francisco, California, United States of America; National Addiction Centre, United Kingdom

## Abstract

**Background:**

In Botswana, an estimated 24% of adults ages 15–49 years are infected with HIV. While alcohol use is strongly associated with HIV infection in Africa, few population-based studies have characterized the association of alcohol use with specific high-risk sexual behaviors.

**Methods and Findings:**

We conducted a cross-sectional, population-based study of 1,268 adults from five districts in Botswana using a stratified two-stage probability sample design. Multivariate logistic regression was used to assess correlates of heavy alcohol consumption (>14 drinks/week for women, and >21 drinks/week for men) as a dependent variable. We also assessed gender-specific associations between alcohol use as a primary independent variable (categorized as none, moderate, problem and heavy drinking) and several risky sex outcomes including: (a) having unprotected sex with a nonmonogamous partner; (b) having multiple sexual partners; and (c) paying for or selling sex in exchange for money or other resources. Criteria for heavy drinking were met by 31% of men and 17% of women. Adjusted correlates of heavy alcohol use included male gender, intergenerational relationships (age gap ≥10 y), higher education, and living with a sexual partner. Among men, heavy alcohol use was associated with higher odds of all risky sex outcomes examined, including unprotected sex (AOR = 3.48; 95% confidence interval [CI], 1.65 to 7.32), multiple partners (AOR = 3.08; 95% CI, 1.95 to 4.87), and paying for sex (AOR = 3.65; 95% CI, 2.58 to 12.37). Similarly, among women, heavy alcohol consumption was associated with higher odds of unprotected sex (AOR = 3.28; 95% CI, 1.71 to 6.28), multiple partners (AOR = 3.05; 95% CI, 1.83 to 5.07), and selling sex (AOR = 8.50; 95% CI, 3.41 to 21.18). A dose-response relationship was seen between alcohol use and risky sexual behaviors, with moderate drinkers at lower risk than both problem and heavy drinkers.

**Conclusions:**

Alcohol use is associated with multiple risks for HIV transmission among both men and women. The findings of this study underscore the need to integrate alcohol abuse and HIV prevention efforts in Botswana and elsewhere.

## Abstract 2

Alcohol use is associated with multiple risks for HIV transmission in men and women. The findings underscore the need to integrate alcohol abuse and HIV prevention efforts in Botswana and elsewhere.

## Editors' Summary

**Background**.**:**

Human immunodeficiency virus (HIV), the cause of acquired immunodeficiency syndrome (AIDS), is most commonly spread through unprotected sex with an infected partner. HIV enters the body through the lining of the sex organs, rectum, or mouth, and destroys immune system cells, leaving the infected person susceptible to other viruses and bacteria. Although HIV education and prevention campaigns emphasize the importance of safe sex in reducing HIV transmission, people continue to become infected by having unprotected sex (that is, not using a condom) with either a nonmonogamous partner or multiple sexual partners, or in situations where they are paying for or selling sex. Research in different populations suggested that heavy alcohol use is associated with risky sexual behaviors. This is because alcohol relaxes the brain and body, reduces inhibitions, and diminishes risk perception. Drinking alcohol may further increase the risk of becoming infected with HIV through its suppressive effects on the immune system.

**Why Was This Study Done?:**

Alcohol abuse is widespread in sub-Saharan Africa where most HIV infections occur and has been associated with risky sexual behaviors. It may therefore be one of the most common, potentially modifiable HIV risk factors in this region. However, research to date has concentrated on the association between alcohol consumption and risky sex in people attending HIV-treatment clinics or recruited at beer halls, and these populations may not be representative of the general population of sub-Saharan Africa. In this study, the researchers have investigated the potential role of alcohol in perpetuating the HIV epidemic by undertaking a population-based study on alcohol use and high-risk sexual behaviors in Botswana. Nearly a quarter of adults are infected with HIV here, and alcohol abuse is also common, particularly in the townships.

**What Did the Researchers Do and Find?:**

The researchers recruited a random cross-section of people from the five districts of Botswana with the highest number of HIV-infected individuals and interviewed all 1,268 participants using a questionnaire. This included general questions about the participants (for example, their age and marital status) and questions about alcohol use, sexual behavior, and knowledge of HIV. Overall, 31% of the men in the study and 17% of the women were heavy drinkers—more than 21 drinks/week for men, 14 for women; a drink is half a pint of beer or a glass of wine. Heavy alcohol use was associated with being male, being in an intergenerational relationship (at least 10 years age difference between partners; intergenerational sex facilitates the continued spread of HIV in sub-Saharan Africa), having had more education, and living with a sexual partner. Among men, those who drank heavily were three to four times more likely to have unprotected sex or multiple partners or to pay for sex than nondrinkers. Among women, there was a similar association between heavy drinking and having unprotected sex or multiple partners, and heavy drinkers were eight times as likely to sell sex as nondrinkers. For both men and women, the more they drank, the more likely they were to have risky sex. The study did not address behavior among same-sex partnerships.

**What Do These Findings Mean?:**

This study indicates that heavy alcohol consumption is strongly and consistently associated with sexual risk behaviors in both men and women in Botswana. Because of the study design, it does not prove that heavy alcohol use is the cause of such behaviors but provides strong circumstantial evidence that this is the case. It is possible that these results may not apply to neighboring African countries—Botswana is unique in being relatively wealthy and in its government being strongly committed to tackling HIV. Nevertheless, taken together with the results of other studies, this research strongly argues for the need to deal with alcohol abuse within HIV prevention programs in sub-Saharan Africa. Strategies to do this could include education campaigns that target both alcohol use and HIV in schools and in social venues, including beer halls. But, stress the researchers, any strategy that is used must consider the cultural and social significance of alcohol use (in Botswana, alcohol use is a symbol of masculinity and high socioeconomic status) and must simultaneously tackle not only the overlap between alcohol use and risky sexual behavior but also the overlap between alcohol and other risk behaviors such as intergenerational sex.

**Additional Information:**

Please access these Web sites via the online version of this summary at http://dx.doi.org/10.1371/journal.pmed.0030392.

- US National Institute of Allergy and Infectious Diseases factsheet on HIV infection and AIDS
- US Department of Health and Human Services information on AIDS
- US Centers for Disease Control and Prevention information on HIV/AIDS
- US National Institute on Alcohol Abuse and Alcoholism patient information on alcohol and HIV/AIDS]
- Aidsmap, information on HIV and AIDS provided by the charity NAM,which includes some information on HIV infections and alcohol
- AVERT information on HIV and AIDS in Botswana

## Introduction

Botswana ranks among the countries with the highest HIV prevalence in the world, with an estimated 24% of adults ages 15–49 infected [1]. Prior research in sub-Saharan Africa has demonstrated that alcohol use and abuse are associated with risky sexual behaviors [2–4], sexually transmitted disease prevalence [4,5], HIV incidence [6], and HIV prevalence [2,7,8]. Sex under the influence of alcohol is associated with both increased HIV prevalence and a greater likelihood of paying for sex [2,4,9]. Alcohol consumption is also associated with a greater likelihood of condom failure and improper use of lubricants with condoms [4]. In a cross-sectional study among men recruited from beer halls in Harare, Zimbabwe, HIV prevalence was shown to increase with increasing levels of alcohol consumption [2]. Simply having visited a beer hall recently has been shown to be associated with HIV prevalence and risky sexual behaviors in several studies from Zimbabwe [10,11]. Intergenerational sexual relations, which are thought to play an important role in the propagation of HIV in Africa [12], are often initiated in drinking establishments [13]. Since alcohol is the most common form of substance abuse in sub-Saharan Africa [14,15], and has been associated with risky sexual behaviors as described above, it may be one of the most common and potentially modifiable HIV risk factors.

Studies to date looking at the association between alcohol consumption and risky sex, however, have largely used potentially biased clinic-based samples or alcohol venue-based sampling strategies [2,4,10,13,16,17]. In addition, risk factors for heavy alcohol use itself with regard to sexual behavior have not yet been adequately characterized. Finally, there are few data on whether the relationship between alcohol and risky sex is the same for men and women, and on the interplay between alcohol, intergenerational relations, and sex exchange. We therefore set out to assess the following in a large, population-based sample covering rural, urban, and semi-urban areas in Botswana: (a) the prevalence and correlates of heavy alcohol consumption; and (b) gender-specific relationships between heavy alcohol use (as a primary independent variable) and a number of HIV transmission risk outcomes, including having unprotected sex with a nonmonogamous partner, having multiple partners, and paying for or selling sex in exchange for money or resources.

## Methods

We conducted a cross-sectional study using a structured survey instrument among a probability sample of 1,268 adults (654 women and 614 men) selected from the five districts of Botswana with the highest number of HIV-infected individuals in November and December of 2004. These five districts cover a population of 725,000 out of a total population of 1.7 million individuals in Botswana.

For the selection of the population-based sample, we used a stratified two-stage probability sample design with the assistance of the Central Statistics Office at the Ministry of Finance and Development Planning in Botswana. The first stage involved the selection of enumeration areas with probability proportional to measures of size, where the measure of size is the number of households in the enumeration area as defined by the 2001 Population and Housing Census. We randomly selected 89 enumeration areas in total, 41 from large urban settlements, 28 from small urban villages, and 20 from rural villages, agricultural lands, and cattle posts. Field researchers then systematically selected households within each enumeration area under the guidance of field supervisors. With a target sample of 1,200 households and an anticipated 85% response rate, 1,433 households were randomly selected. We used random number tables within each household to select one adult member that met our inclusion criteria, and up to two repeat visits were made. No replacements were made if participants could not be contacted after two visits.

### Study Procedures

We pilot-tested our survey instrument among 20 individuals from Gaborone, Botswana's capital. A four-day training course on survey implementation was given to 25 qualified local researchers. To check the accuracy of translation, we translated survey instruments and consent forms into Setswana and then back-translated them into English. All interviews lasted 45–60 minutes, and were conducted in either English or Setswana in a private setting. Written consent was obtained from all study participants. Participants were excluded if they were younger than 18 years or older than 49, did not speak either English or Setswana, had cognitive disabilities, were not a resident of Botswana, or if there was inadequate privacy to conduct the interviews. The UCSF Human Subjects Committee and the Botswana Ministry of Health Research and Development Committee approved all study procedures.

### Measures

Domains of inquiry for our surveys were based on previously validated instruments [18–20], and included demographic characteristics, use of alcohol, sexual practices (including HIV risk behaviors), history of HIV testing, symptoms of depression, and measures of health care access and utilization.

To measure alcohol use, we asked participants to indicate the number of days per week that they drank alcohol, as well as the number of drinks per day on the days that they drank. From this information, we then calculated the number of drinks per week. Alcohol use was defined in four categories: (a) no drinking; (b) moderate drinking (1–7 drinks/week for women and 1–14 for men), (c) problem drinking (8–14 drinks/week for women, 15–21 for men) [19]; and (d) heavy drinking (>14 drinks/week for women, >21 for men).

To measure HIV knowledge, we adapted questions from the UNAIDS General Population Survey and the DHS AIDS module. Individuals were scored as having correct HIV knowledge if they were able to identify the two most common modes of HIV prevention in Botswana (consistent condom use and abstinence) as specified in the UNAIDS knowledge indicator scoring system [18].

Symptoms of depression were measured using the 15-item Hopkins Symptom Checklist for Depression (HSCL-D) [20], which has been validated previously in a number of international settings in Africa and elsewhere [21]. A score of 1.75 or above corresponds to a diagnosis of depression according to this measure.

### Analysis

Our overarching analysis plan was to assess the potential role of alcohol use in perpetuating the HIV epidemic. Descriptive statistics were used to examine prevalence of alcohol consumption and frequency of alcohol use before sex over the past 12 months among men and women in our sample. Pearson's Chi^2^ tests were used to compare men and women on demographic and behavioural variables of interest. To determine correlates of alcohol use, we used both ordinal logistic regression with the four categories of alcohol use listed above, as well as multivariate logistic regression with heavy drinking as the primary outcome. Since results were similar using the two strategies, results of logistic regression only are presented for simplicity.

We then used multivariate logistic regression to examine the impact of alcohol use as a primary independent variable (defined according to the four categories listed above) on several sexual risk outcomes. These analyses were stratified by gender, since gender is well known to modify sexual risk-taking, and key outcome measures differed for men and women (e.g., paying for sex versus receiving money for sex). Our primary transmission risk outcome was unprotected sexual intercourse with a nonmonogamous partner over the past month. Additional outcomes included (a) having multiple partners over the past 12 months; (b) exchanging sex for money, or other resources over the past 12 months (analyses presented for women only, because very few men reported this behaviour); and (c) paying for or providing resources for sex over the past 12 months (analyses presented for men only,because very few women reported this behaviour). We did not ask specifically about sexual orientation.

Specific variables controlled for in our analysis were selected based on prior literature and theory [2–4,6,7,9,10,13,17,22,23] and included: (a) age (continuous per year); (b) sex (male or female); (c) income (<1,000 or ≥1,000 Pula/month [where 1 Pula was equivalent at the time of the study to approximately US$0.25]); (d) education (did not complete high school or had at least a high school education); (e) residence type (rural, urban, or urban village); (f) marital status (married, living with partner, or other); (g) knowledge surrounding HIV/AIDS (correct or incorrect); (h) symptoms of depression (continuous measure with score ranging from 1.0 to 4.0); (i) frequency of visits to a medical provider (less than one, one or two, or three or more visits/year); (j) history of testing for HIV (yes or no); (k) intergenerational relationships, defined as having had sexual relations with a partner ten or more years older or younger in the past year [24] (yes or no); (l) lack of control in sexual decision-making in the previous 12 months (participants were included in this category if they believed that their partner usually or always made the decision as to whether or not to have sex); and (m) health status (very good or good versus fair or poor).

All data were analyzed with STATA statistical software version 8.0 [25]. Categorical variables were compared with Chi^2^ tests and continuous variables by t-tests. Regression diagnostic procedures yielded no evidence of multicolinearity or overly influential outliers in any of the models. Logistic regression data are supplemented by descriptive statistics on the views of respondents about the key barriers to condom use, and on the principal factors that put men and women at risk for HIV in Botswana.

## Results

### Participant Characteristics

A total of 1,433 individuals were randomly selected. Of these, 165 people were excluded from the study sample: 46 (3.2%) were unavailable after two repeat visits, 78 (5.4%) refused or did not meet criteria, and 41 (2.9%) were unable to complete the interview. Among the 1,268 (88.5%) respondents that completed the survey, 654 (52%) were female. Additional demographic and behavioral characteristics of the study population are shown in Table 1. Criteria for problem drinking were met by 39% (*n* = 238) of men, and of these 79% (*n* = 188) also met criteria for heavy drinking (Table 1). Among women, 25% (*n* = 163) met criteria for problem drinking, and of these, 69% (*n* = 113) were heavy drinkers. Drinking alcohol before sex in the past year on a regular or semiregular basis was reported by 39% of men and 20% of women. While 38% of respondents reported having had unprotected sex over the past year, only 12%–13% of both men and women said that they had unprotected sexual intercourse with a nonmonogamous partner during the past month. Over the previous year, 46% of men and 28% of women had multiple partners. In semistructured questions, 45% of participants identified alcohol use as the single most important factor that makes both men and women vulnerable to HIV in Botswana.

**Table 1:**
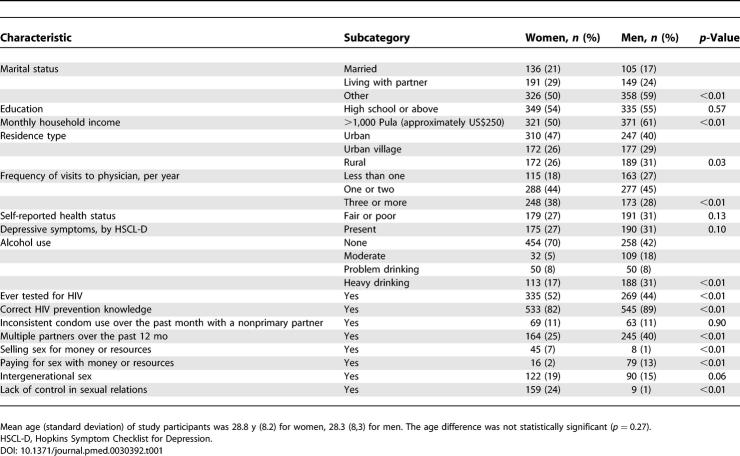
Baseline Characteristics of Respondents, Botswana, 2004 (*n* = 1,268)

### Demographic and Behavioral Correlates of Heavy Alcohol Consumption

Because there were few differences in correlates of alcohol use by gender, data are presented for the sample as a whole (Table 2). In adjusted analyses, people with a history of intergenerational sexual relationships had nearly three times the odds of heavy alcohol use (95% confidence interval [CI], 1.82 to 3.69). Males also had nearly three times the odds of heavy drinking when compared to females (95% CI, 1.89 to 3.58). People living with a sexual partner had two times the odds of heavy drinking (95% CI, 1.43 to 2.81), and individuals with more education had approximately 40% higher odds of heavy drinking (95% CI, 1.05 to 2.00).

**Table 2:**
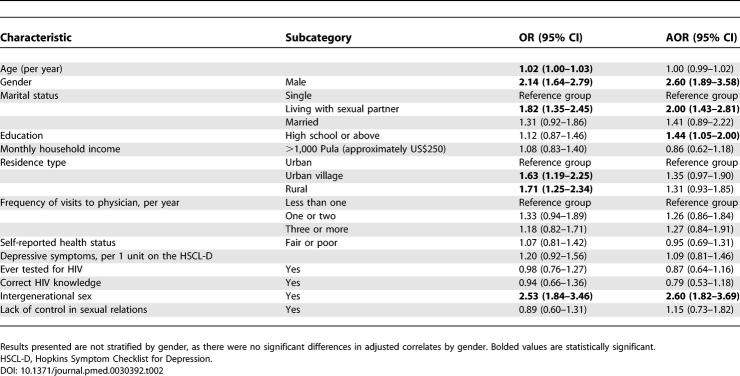
Unadjusted and Adjusted Associations with Heavy Drinking, Botswana, 2004 (*n* = 1,196)

### Correlates of Unprotected Sexual Intercourse

Unadjusted and adjusted correlates of unprotected sex with a nonmonogamous partner are displayed in Table 3, stratified by gender. After potential confounding factors were adjusted for, men and women who screened positive for heavy drinking had over three times the odds of reporting a history of unprotected sex with a nonmonogamous partner. There was a dose-response relationship between alcohol consumption and unprotected sex for both genders, in that heavy drinkers (>14 drinks/week for women, >21 for men) had higher odds of unprotected sex than did problem drinkers (8–14 drinks/week for women, 15–21 drinks/week for men), who in turn had had higher odds of unprotected sex compared to moderate drinkers. People who consumed no alcohol were at lowest risk for unprotected sex. A history of intergenerational sex was associated with over three times the odds of engaging in unprotected sex for women and nearly three times the odds of unprotected sex for men. For women only, older age and greater HIV/AIDS prevention knowledge were associated with lower odds of unprotected sex.

**Table 3:**
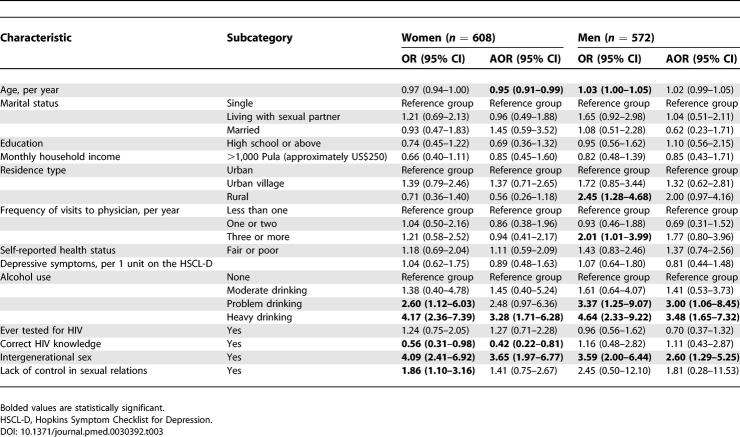
Unadjusted and Adjusted Associations of Unprotected Sex with Nonprimary Partner over Past Month by Gender; Botswana, 2004

The cited barriers to using condoms differed for men and women when compared using Pearson's Chi^2^ tests. More men cited the belief that condoms decrease sexual pleasure (69% of men versus 46% of women; *p* < 0.001), and that condoms are inconvenient (29% of men versus 15% of women; *p* < 0.001). More women claimed that they did not use condoms because their partners usually refused (53% of women compared to 13% of men; *p* < 0.001), or because they lacked control in sexual decision-making (22% of women versus 7% of men; *p* < 0.001). Fewer than 6% of both men and women cited not knowing how to use condoms, not having condoms available, believing that condoms do not prevent AIDS, or not being able to afford condoms as significant barriers to condom use.

### Correlates of Having Multiple Sexual Partners

Problem drinking, heavy drinking, and intergenerational sex were also strongly associated with having multiple partners for both men and women in unadjusted and adjusted analyses (Table 4). In addition, lack of control in sexual relationships was significantly correlated with having multiple partners for both genders. Living with a sexual partner was associated with having multiple partners for both genders in unadjusted analyses and for women only in adjusted analyses. Having more depressive symptoms was correlated with having multiple partners for both genders in unadjusted analyses, and for women only in adjusted analyses. For men only, having had an HIV test was associated with a lower odds of having multiple partners, and more frequent health care contacts was correlated with a higher odds of having multiple partners. In addition, for men only being married was associated with lower odds of having multiple partners, and having greater HIV knowledge was associated with higher odds of having multiple partners.

**Table 4:**
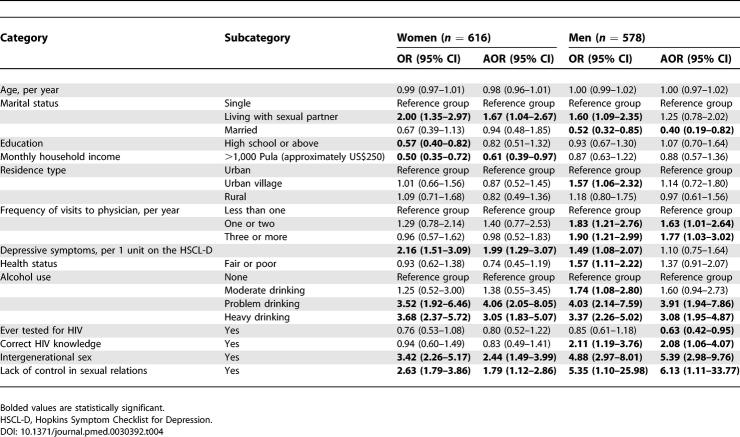
Bivariate and Adjusted Odds of Having Multiple Sexual Partners over the Past 12 Months; Botswana, 2004

### Correlates of Sex Exchange

Alcohol use was also strongly associated with both paying for and providing sex for resources when adjusting for possible confounders (Table 5). Men who were moderate drinkers had nearly three times the odds of paying for sex with money or other resources compared to nondrinkers, and men who were heavy drinkers or problem drinkers had approximately four to five times the odds of paying for sex compared to nondrinkers. Women who were problem drinkers or heavy drinkers had over eight times the odds of providing sex in exchange for money or resources compared to nondrinkers. Men engaging in intergenerational sex had nearly four times the odds of paying for sex, and women engaging in intergenerational sex had nearly six times the odds of sex exchange in adjusted analyses. Additional factors associated with paying for sex among men in multivariate analyses included more symptoms of depression, and fair or poor self-reported health status. Additional adjusted correlates of exchanging sex for resources among women included living with a sexual partner and lacking control in sexual relationships.

**Table 5:**
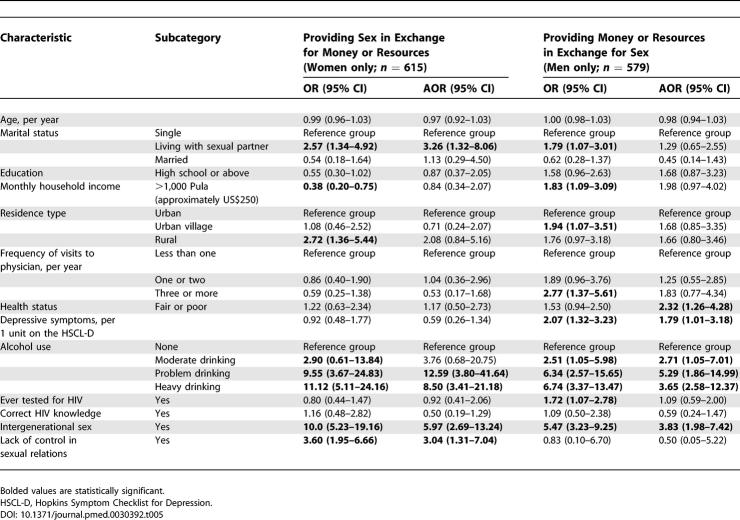
Bivariate and Adjusted Odds of Engaging in Sex Exchange; Botswana, 2004

## Discussion

To our knowledge, this is one of the first population-based assessments to explore the association between alcohol use and a number of high-risk sexual behaviors in sub-Saharan Africa. We found that nearly 40% of men, and over 25% of women reported problem drinking, and the majority of these also met criteria for heavy drinking. High levels of alcohol use and dependence have similarly been found in neighboring countries, including South Africa and Zimbabwe [4,11,26]. Approximately 40% of men, and 20% of women in our study claimed to drink regularly before sex. Our findings that men and those of higher education levels were more likely to drink are consistent with the cultural depiction of alcohol as a symbol of masculinity and of higher socioeconomic status in Botswana [27].

Heavy alcohol consumption was a strong and consistent correlate of all sexual risk behaviors examined for both men and women, including unprotected sexual intercourse with a nonmonogamous partner, having multiple partners, and paying for or exchanging sex for money or other resources. Parallel to findings from our multivariate regression analyses, 45% of participants identified alcohol use as the single most important HIV risk factor in semistructured questions. Our results from this large probability sample of adults in Botswana reinforce the findings from a number of smaller studies that alcohol use is strongly associated with a number of risky sexual behaviors in sub-Saharan Africa [2–4]. For example, in a study among 324 male beerhall patrons in Zimbabwe, Fritz et al. demonstrated that the number of days of alcohol consumption was correlated with the number of episodes of unprotected sex with a casual partner and with episodes of paying for sex. In another study by Simbayi et al. among 149 men and 72 women receiving sexually transmitted disease services in Capetown, South Africa, participants with more problematic drinking had significantly more sexual partners over the previous month, were more likely to have received money for sex, and were more likely to have a history of sexually transmitted diseases. These results were not stratified by gender. Dunkle et al. showed that problem drinking was associated with significantly higher odds of sex exchange in women attending antenatal clinics in Soweto, South Africa [17]. Our data extend this previous research by demonstrating that these associations hold in a population-based study, that the relationships between alcohol use and risky sexual practices are similar in both men and women, and that there is a dose-response relationship between alcohol use and unprotected sex as well as other risky sexual practices among both genders. Our findings, in conjunction with those of others, strongly argue for the need to target alcohol use and abuse in HIV prevention programs. While causality can not be determined from a cross-sectional study, the consistency of results across many studies (including the reported associations between alcohol use and incident HIV infection [6]), the dose-response relationship between alcohol and risky sex, the strength of the associations, and the biologic plausibility all suggest that alcohol use is in fact a cause rather than a consequence of risky sexual behavior.

To date few policies have been implemented in Botswana and elsewhere in Africa to address the strong overlap between alcohol use and HIV. A study by Fritz et al. has shown that it is methodologically feasible and culturally appropriate to carry out HIV interventions in Zimbabwe beer halls [2]. Additional risk reduction strategies that could be considered include educational campaigns targeting alcohol and HIV in schools and other social venues, interventions that limit alcohol licenses or increase taxes on alcohol, and the bolstering of programs for the prevention, treatment, and rehabilitation of alcohol abuse. Alcohol use in Botswana and elsewhere in Africa has deep-seated cultural and social meanings related to social status, gender identity, and family and communal structures [27] that must be taken into account in the design of effective alcohol reduction strategies.

The most significant correlates of risky sexual behavior were similar for men and women. In addition to alcohol use, intergenerational sex was strongly and consistently associated with all risky sex variables for both men and women, and was also strongly associated with heavy drinking. Previous qualitative studies have shown that intergenerational and transactional sexual relationships are often initiated in drinking establishments [13]. Intergenerational sex can be a major contributor to propagating and sustaining HIV in sub-Saharan Africa, as it can account for substantial amounts of HIV transmission between different age groups [12]. In view of the strong overlap between risky alcohol use, intergenerational sex, and sexual risk-taking, our results attest to the need for multidimensional approaches in HIV prevention programs that simultaneously target a number of high-risk practices. For example, reinforcing or raising the drinking age may also be effective at decreasing intergenerational sex, and programs to promote economic independence for women may help overcome the financial dependency that promotes intergenerational sex and sex exchange [28].

We found that lack of control in sexual relationships was associated with having multiple partners for both men and women, and with sex exchange for women. Not surprisingly, women were significantly more likely than men to report lack of control in sexual relationships, and were also more likely to consider lack of control in sexual relationships and a partner's refusal to use condoms as key barriers to condom use. These findings are consistent with studies showing that lower relationship control and forced sex for women are associated with both inconsistent condom use [29] and higher HIV seroprevalence [30]. Higher negotiating power within sexual relationships and economic independence were also found to be positively associated with condom use in a small cross-sectional study among 71 women in Gaborone, Botswana [22]. HIV prevention programs are more likely to be effective if they address the pervasive gender discrimination that helps to perpetuate the spread of HIV in Botswana and elsewhere in Africa [30]. Interventions should also target the complex inter-relationships between alcohol use and gender economic imbalances.

A few important gender differences were notable in correlates of sexual risk-taking. For example, older age was associated with lower odds of unprotected sex for women, and the reverse trend was apparent for men. This is consistent with statistics on HIV prevalence in Botswana (with evidence that HIV prevalence is three times as high among younger women than younger men [31]) as well as with the aforementioned finding that intergenerational sex is associated with a greater likelihood of unprotected sex since these relationships are most common between younger women and older men. Men but not women with more frequent contact with health providers were more likely to have multiple partners, and men who reported poor health were more likely to pay for sex. It is more likely that frequent contact with health providers and poor health are a consequence and not a cause of these risky sexual practices. HIV testing was associated with a lower likelihood of having multiple partners for men but not women. This may be because men are in a better position to change their sexual behavior after HIV testing compared to women, who may lack control over sexual negotiation, as discussed above. Greater symptoms of depression were associated with having multiple partners for women and paying for sex for men. This is consistent with studies in developed countries that have shown a strong link between depression and high-risk sexual behavior [32,33], but no studies to our knowledge have previously examined this association in an African context. In view of the high prevalence of depressive symptoms reported in this study (nearly 30% of participants screened positive using the Hopkins Symptom Checklist), it is important to further explore depression as both a possible cause and consequence of the high-level of HIV transmission in sub-Saharan Africa.

In addition to the cross-sectional design, a number of limitations affect interpretation of our results. Self-reporting may introduce bias, because it can be influenced by social desirability. To minimize self-reporting bias, we did not ask about HIV status, assured confidentiality and privacy in all interviews, and carefully trained interviewers on asking sensitive questions in a nonjudgmental fashion. While risky sexual practices may have been underreported, this would not necessarily affect the associations we found between risky sex and alcohol use as well as other covariates. Another limitation was that our measures on alcohol use did not address either alcohol dependence or impairment in social functioning. Findings would have been strengthened if we used the internationally validated WHO Alcohol Use Disorders Identification Test (AUDIT) as a screening tool for alcohol use. Future validation studies of Western standardized alcohol abuse screening tools in African settings should pay careful attention to the fact that consumption of home-brewed drinks may be more difficult to quantify. Finally, Botswana has a number of unique features that may limit generalizability to neighboring African countries, such as its relatively high per capita income, comparatively extensive health care infrastructure, strong donor involvement, and strong government commitment to combating HIV. Nonetheless, similar results from studies in other African countries [2,4,10,13] strongly support these findings and their applicability elsewhere in Africa.

### Concluding Remarks

In summary, we found a very high prevalence of heavy alcohol consumption in a large probability sample of rural and urban individuals in Botswana, consistent with the results of previous venue-based studies elsewhere in Africa. We demonstrated a strong and consistent relationship between heavy alcohol use and a number of risky sexual behaviors among both men and women, including the important link between sex exchange and heavy alcohol use. This study also confirms the associations between different risky sexual practices and intergenerational sex, and also points to other important correlates of risky sex that have not been previously studied in sub-Saharan Africa, such as symptoms of depression. The findings in this study underscore the importance of integrating policies on alcohol abuse in HIV prevention efforts in Botswana and elsewhere, and attest to the need for multipronged approaches to HIV prevention that simultaneously address the overlap of risk behaviors as well as some of the social, cultural, and structural factors that help fuel the HIV epidemic.

## Abbreviation

AOR: adjusted odds ratio
